# The Observer Introductory

**Published:** 1881-04

**Authors:** 


### THE OBSERVER'S INTRODUCTORY FOR 1881.

We are glad to learn that the *American Observer* has such a profund respect for the opinions and writings of Hahnemann, and that its editor ranks himself among the followers of that great man. That is well; for we believe likewise in the teachings of the master. But we are sorry to see the bitter and intolerant spirit the *Observer* entertains against a society recently formed, called the International Hahnemann Association. Against that society it makes a violent onslaught, without a particle of justice or reason; charging it with advocating high potencies and forming a separate organization and advocating isopathy.

The *Observer* knows that the International Hahneman Association believes in the efficiency of high potencies, and in their efficacy to cure disease, and that its members therefore created a separate organization that they might more efficiently carry out that object, and thereby induce the practice of pure homœopathy. The *Observer*, however, "with all its strength and heart" stands opposed to the International Hahnemann Association, and its organ, The Homœopathic Physician.

Its title seems in the eyes of the *Observer* "monstrous," an

"assumption little short of sacrilege and clearly libelous." How it can apply such terms with sense or propriety to a journal devoted strictly to homœopathic practice we can not understand. The only solution we can give is, that the prejudices of the head, and the gall of the heart have given rise to a bilious condition and imparted a cloudy, false coloring to the vision.

The *Observer* has for a long time, as it declares, advocated high and low potencies, and contended for the right of the homeopathic physician to use the whole range of remedies, from the drug to the mother-tincture, as in his judgment appears to be best. With this avowed liberality of opinion and practice, in the same breath, with great inconsistency, the *Observer* denies the right of physicians to associate themselves together, having one mind to carry out the application of the law of *Similia Similibus.* Forgetting the liberality of its declarations, the *Observer* opposes them "with all its strength and all its heart," and in the hate of its heart, and in the use of all its strength, it declares they have violated the law as laid down by Hahnemann, because they do not think with it, but hold their own views of a truth as proclaimed by Hahnemann.

We shall not be able to give more room this month to the "Introductory" of the *Observer*; but each month we shall take up in their order the grounds of opposition taken by the *Observer* against the International Hahnemann Association and THE HOMŒOPATHIC PHYSICIAN.

"INTERNATIONAL."

